# Demonstration of Non-Gaussian Restricted Diffusion in Tumor Cells Using Diffusion Time-Dependent Diffusion-Weighted Magnetic Resonance Imaging Contrast

**DOI:** 10.3389/fonc.2016.00179

**Published:** 2016-08-02

**Authors:** Tuva R. Hope, Nathan S. White, Joshua Kuperman, Ying Chao, Ghiam Yamin, Hauke Bartch, Natalie M. Schenker-Ahmed, Rebecca Rakow-Penner, Robert Bussell, Natsuko Nomura, Santosh Kesari, Atle Bjørnerud, Anders M. Dale

**Affiliations:** ^1^The Interventional Centre, Oslo University Hospital, Oslo, Norway; ^2^Department of Circulation and Medical Imaging, Norwegian University of Science and Technology, Trondheim, Norway; ^3^Department of Radiology, University of California San Diego, La Jolla, CA, USA; ^4^Department of Neurosciences, University of California San Diego, La Jolla, CA, USA; ^5^Department of Physics, University of Oslo, Oslo, Norway

**Keywords:** MRI, xenograft tumor model, DWI, restricted diffusion, diffusion time-dependence, glioblastoma multiforme, cancer

## Abstract

The diffusion-weighted magnetic resonance imaging (DWI) technique enables quantification of water mobility for probing microstructural properties of biological tissue and has become an effective tool for collecting information about the underlying pathology of cancerous tissue. Measurements using multiple *b*-values have indicated biexponential signal attenuation, ascribed to “fast” (high ADC) and “slow” (low ADC) diffusion components. In this empirical study, we investigate the properties of the diffusion time (Δ)-dependent components of the diffusion-weighted (DW) signal in a constant *b*-value experiment. A xenograft gliobastoma mouse was imaged using Δ = 11 ms, 20 ms, 40 ms, 60 ms, and *b* = 500–4000 s/mm^2^ in intervals of 500 s/mm^2^. Data were corrected for EPI distortions, and the Δ-dependence on the DW-signal was measured within three regions of interest [intermediate- and high-density tumor regions and normal-appearing brain (NAB) tissue regions]. In this study, we verify the assumption that the slow decaying component of the DW-signal is non-Gaussian and dependent on Δ, consistent with restricted diffusion of the intracellular space. As the DW-signal is a function of Δ and is specific to restricted diffusion, manipulating Δ at constant *b*-value (cb) provides a complementary and direct approach for separating the restricted from the hindered diffusion component. We found that Δ-dependence is specific to the tumor tissue signal. Based on an extended biexponential model, we verified the interpretation of the diffusion time-dependent contrast and successfully estimated the intracellular restricted ADC, signal volume fraction, and cell size within each ROI.

## Introduction

The diffusion-weighted magnetic resonance imaging (DWI) technique enables probing of microstructural properties of biological tissue and is frequently used as an imaging biomarker for the detection and quantification of the underlying pathology of cancerous tissue.

The diffusion-weighted (DW) signal from a biological medium using pulsed-gradient spin echo measurements is commonly modeled with the following equation:

(1) $$S\left(b\text{,}TE\right)=S_{\text{0}}\text{exp(}-{\text{TE/T}}_{\text{2}}\text{)exp(}-b\cdot \text{ADC)}$$

where *T*_2_ is the spin–spin relaxation time, ADC is the apparent diffusion coefficient of the medium, and *S*_0_ is the MR signal with no applied diffusion- or *T*_2_-weighting. TE is the echo time of the experiment and *b* is the diffusion signal weighting given by (1)

(2) $$b=\lambda^{\text{2}}G^{\text{2}}\delta^{\text{2}}\left(\Delta -\frac{\delta }{\text{3}}\right)$$

here, γ is the gyromagnetic constant, *G* is the diffusion gradient amplitude, δ is the gradient duration, and Δ is the diffusion time given by the separation time between the two applied diffusion gradients.

Diffusion-weighted magnetic resonance imaging and corresponding ADC maps is considered a highly promising imaging biomarker of high-grade tumor tissue as it allows, for characterization of tumor pathology demonstrating, a negative correlation between tumor cell density and ADC (2, 3). However, despite the high cellularity of these tumors, the ADC has limited specificity as concomitant edema and tumor-related necrosis has shown to increase the ADC values and thereby opposing the reduced ADC associated with the tumor (4, 5).

Measurements using multiple *b*-values have indicated a biexponential signal attenuation, ascribed to “fast” (high ADC) and “slow” (low ADC) diffusion components within a tissue (6–10). The “fast” diffusion component is normally assumed to correspond to the hindered diffusion of free water molecules colliding with cellular borders with a Gaussian diffusion displacement probability where mean squared displacement is given by <*r*^2^> = 6ADCΔ (11). As hindered ADC of the brain is related to the intrinsic diffusion coefficient of extracellular water (*D*_ex_) and the tortuosity of the tissue (λ) by ADC_h_ = *D*_ex_/λ^2^, where 1 < λ < 1.8 (about 1.5 for healthy brain tissue) (12–15) and *D*_ex_ ~ 3 μm^2^/ms (4), DW-signals from hindered compartments are strongly attenuated at high *b*-values (*b* > λ^2^/*D*_ex_). The “slow” diffusion component exhibits a lower ADC (slower signal decay) compared to the fast, hindered ADC and is most commonly associated with restricted diffusion of spins (water) trapped within confined compartments (6, 10, 13, 16, 17). However, the slow diffusion component has also been proposed to represent increased tortuosity or slower intrinsic diffusivity, which can also be associated with decreased ADC (18, 19), hence the basic mechanism underlying the slow ADC still remains unclear.

There is a fundamental difference in diffusion physics between water molecules that are hindered (and free) versus restricted. At the time scales measured by most DWI experiments (Δ ~ 20–60 ms), hindered and free water molecules follow a Gaussian displacement probability distribution, whereas restricted water molecules demonstrate non-Gaussian displacement probability due to physical restrictions imposed on intracellular water molecules by plasma membranes. The mean square distance traveled by intracellular restricted spins, <*r*^2^>, is therefore a function of the size (*d*) and shape of the cellular compartments as well as the intrinsic diffusivity of intracellular water (*D*_intra_) (13, 20–22). At the “short diffusion time regime” (i.e., *d*^2^ ≫ 2*D*_intra_Δ), diffusion will be largely unrestricted as only spins near the cellular membranes will experience the confining borders. The measured ADC is then dictated by the *D*_intra_ of the medium, and the total DW-signal follows monoexponential signal decay (Eq. 1) consistent with Gaussian diffusion behavior. When increasing Δ, a larger portion of spins will come in contact with the plasma membrane, resulting in an increased signal contribution from restricted diffusion and decreasing ADC. In this diffusion time regime, the total DW-signal no longer follows a monoexponential signal decay but, instead, approaches a biexponential signal decay (22). For clinical relevant Δ (50–100 ms), the diffusion time is short relative to the exchange rate between intra- and extracellular compartments (23) but long enough for intracellular spins to repeatedly come in contact with the plasma membrane (i.e., *d*^2^ ≫ 2D_intra_Δ). In this time regime, intracellular diffusion approaches a restricted “fill-up” regime where ADC is low (e.g., ADC ~ 0.1 μm^2^/ms at Δ = 60 ms) (22, 24), and the restricted DW-signal remains present even at high *b*-values (7, 13). It is important to note the difference between restricted diffusion, as defined classically as diffusion of water confined within cellular compartments, and “restriction” as reported at short Δ when spins are observed to bounce off cellular membranes in oscillating gradient experiments (25, 26). The latter has little effect on the measured ADC of clinical studies (26, 27), and, in this paper, we will use the definition of restricted diffusion as the clinical relevant fill-up of confined space.

Numerous previous studies have modeled the biexponential signal behavior with the assumption that the slow component of the DW-signal attenuation is a consequence of intracellular restriction (6, 20, 28–30). When utilized in cancer imaging, studies show improved tumor contrast-to-noise and reduced sensitivity to extracellular edema when comparing the slow ADC to standard (extracellular) ADC DWI (31–34). Restriction spectrum imaging (RSI) is an advanced DWI modeling technique that isolates the slow diffusion component by separating and removing signal from hindered diffusion. The technique offers a direct measure of tumor cellularity and the results suggest that the restricted water fraction is specifically sensitive to tumor presence, particularly high-grade tumors (35). In this study, we explore the non-Gaussian nature of the DW-signal in order to verify the relationship between the underlying diffusion mechanism of the tissue and the measured slow ADC. As the DW-signal as a function of Δ is specific to restricted diffusion, manipulating Δ at constant *b*-value (cb) provides a complementary and direct approach for separating the restricted from the hindered diffusion component. It follows that subtracting diffusion data acquired at long and short Δ using this setup will isolate the Δ-dependent non-Gaussian restricted water signal by removing signals from the free and hindered water pools. Thus, using this approach, we are able to determine the origin of the slow diffusion component of the DW-signal. We demonstrate the Δ-dependence of the DW-signal in a human glioblastoma (GBM) xenograft mouse model using a modified *in vivo* imaging protocol that manipulates both *b*-values and Δ. We further show that our findings are supported by a biexponential signal fit, dependent on Δ.

## Materials and Methods

This animal study was granted ethical approval through Institutional Animal Care and Use Committee (IACUC) and performed according to their guidelines.

### Xenograft Model

For intracranial glioma xenograft experiments, patient-derived glioma stem cell like cells (cell line designated GBM4) were collected and re-suspended at 3 × 10^5^ cell/3 μL in HBSS solution and placed on ice until injection (36, 37). Five anesthetized 6-week-old female NSG mice (stock number 005557, Jackson laboratory) were placed on a stereotactic frame with ear bars. A hole was drilled 2 mm lateral and 1.5 mm anterior to the Bregma. 3 × 10^5^ cells in 3 μL volume were then injected using a Hamilton syringe and needle 2 mm below the skull into the striatum of the brain. Mice were then monitored periodically and euthanized for imaging when neuropathological symptoms were evident. Based on the quality of the MRIs and the histology, one suitable mouse was found suitable for the analysis.

### Magnetic Resonance Imaging

The magnetic resonance imaging (MRI) scans were performed on a 7 T (20 cm bore) Bruker Biospin animal magnet (Bruker Instruments, Ettlingen, Germany) located at the University of California, San Diego, CA, USA, Center for functional-MRI, using a 72 mm ID linear birdcage volume resonator (Bruker) with PIN diode-based active decoupling capability in combination with a two channel local receive array coil (Rapid Biomed).

The imaging protocol included two structural TurboRARE *T*_2_-weighted with echo TE/TR = 36 ms/5934.8 ms, field of view (FoV) = 1.8 cm, and matrix resolution = 166 × 125, one with a high slice resolution of slice thickness = 0.2 mm (*T*_2,high_) and number of slices = 50 and one set with slice resolution (*T*_2,low_) according to that of the DW images of 10 slices of slice thickness = 1.0 mm. The DWI was performed using a spin echo echo-planar imaging sequence with 8 different *b*-values of 500, 1000, 1500, 2000, 2500, 3000, 3500, and 4000 s/mm^2^ at fixed gradient duration δ = 6 ms, and one *b* = 0 acquisition and 6 non-collinear diffusion gradient directions (used for averaging), TE/TR = 79.6 ms/5000 ms, FoV = 1.8 cm, matrix resolution = 64 × 64, number of slices = 10, and slice thickness = 1 mm. The experiment was repeated for diffusion times, i.e., the timing between the two diffusion weighted gradient pulses, of Δ = 11 ms, 20 ms, 40 ms, and 60 ms. Subsequent scans acquired with the different Δ were run with constant receiver gain. In addition, one acquisition of a *b* = 0 volume using a reverse phase encoding direction was acquired for the correction of intensity distortions associated with the *B*_0_ field (38).

### Histology Preparation and Image Acquisition of Immunofluorescent- and H&E-Stained Samples

Histology slides were obtained to quantify relative cell density measurements within specified regions of interest (ROIs) in order to support any observed difference in the DWI signal. The histology slides and DWI slides were not coregistered due to the limited resolution of the DWIs, and the regions were thus only matched for quantitative verification.

Immediately after the scans, the mouse brain was removed, fixed in 4% paraformaldehyde overnight at room temperature, dehydrated with ethanol, and then embedded in paraffin. 5-μm thick mouse brain sections were sliced and placed on glass slides and stained with Hematoxylin & Eosin (H&E). For the immunofluorescence (IF) staining, the 5-μm affixed sections were treated with 1× Trilogy™ pretreatment solution (1:20 dilution; Cell Marque, Rocklin, CA, USA) using the manufacturer-described steamer method in order to remove paraffin and prepare the tissue for epitope retrieval. The tissue was then blocked with 10% Donor Goat Serum (GS; Gemini Bio Products, Sacramento, CA, USA) in 1× TBS for 1 h and then quickly rinsed twice with TBS. Each sample was incubated with 300 μL of 5 μg/mL mouse monoclonal anti-CD56 antibody (1:200 dilution; N-CAM clone 123C3, Invitrogen, Carlsbad, CA, USA) (39) for 18 h at RT on an orbital shaker. Each slide was then quickly rinsed with TBS twice and incubated with 300 μL of 2 μg/mL Alexa Fluor^®^ 568 goat anti-mouse H + L (1:1000 dilution; Invitrogen, Carlsbad, CA, USA) for 26 h at RT on an orbital shaker. The cell nuclei were stained using ProLong^®^ Gold antifade reagent with DAPI (Invitrogen, Carlsbad, CA, USA).

The IF images were acquired with a Keyence BZ-X710 all-in-one fluorescence microscope (Osaka, Japan) using DAPI and Texas Red filter cubes with an S Plan Fluor 0.45NA ELWD 20× objective lens. The H&E images were acquired using transmitted light and a 20×/0.75 N.A. lens on an Olympus IX81 microscope. Exposure time and White Balance were calculated automatically by the system. All scans were acquired at high resolution (0.37744 μm/pixel).

### Image Analysis

All imaging data were processed offline in Matlab [MathWorks, version 8.1.0 (R2013a)] using in-house developed software.

**Figure 1:**
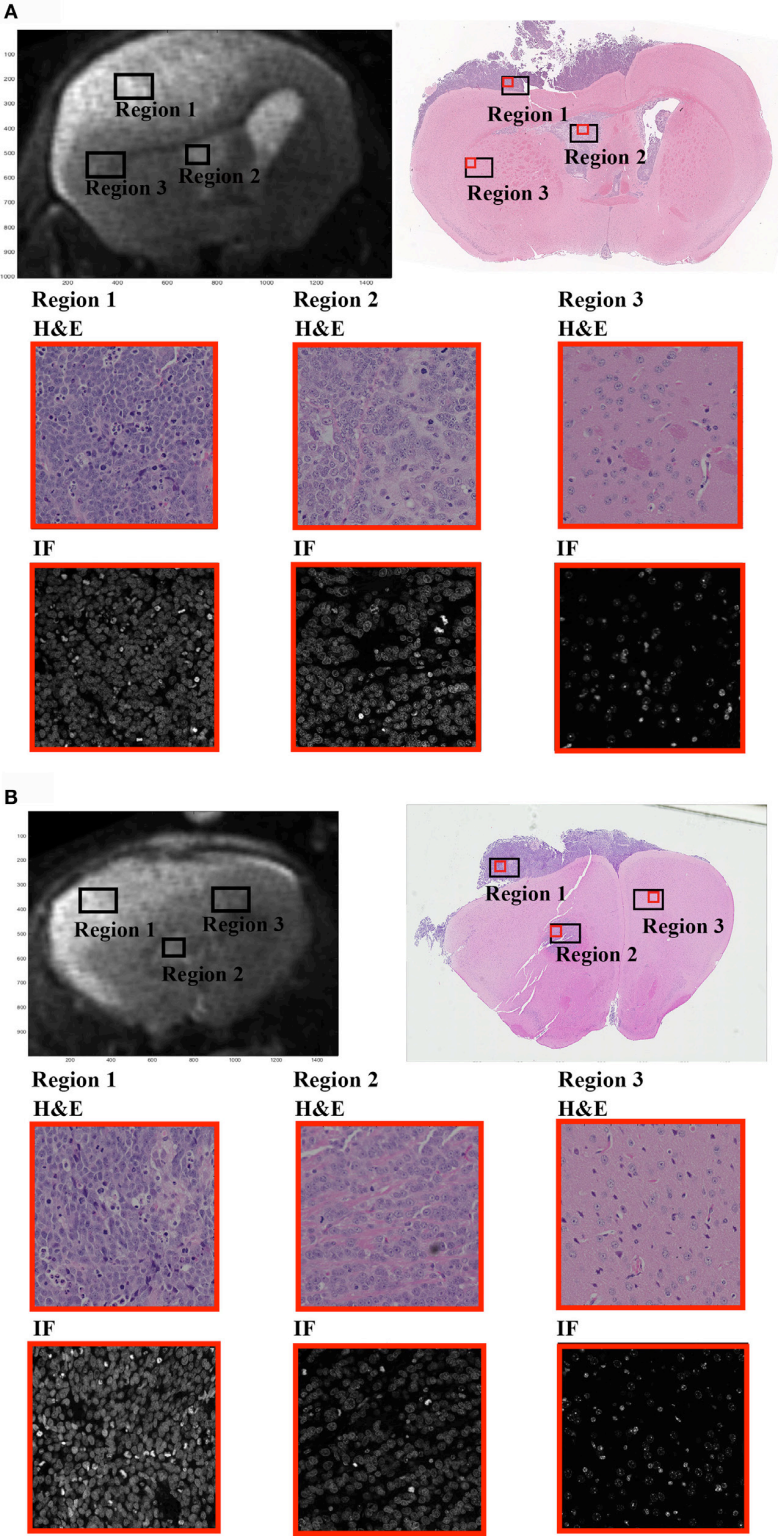
**In the presented study, we have investigated the diffusion time (Δ)-dependence on the DW-signal within three regions of interest (ROIs) selected from MRI brain images of a single GBM xenograft mouse**. **(A,B)** Display the two MRI slices selected for the study (upper left) along with the corresponding H&E-stained sections (upper right). Region 1 represents the region of high tumor cell density (nuclei coverage ~50%), region 2 of intermediate tumor cell density from within the ventricles (near injection site, nuclei coverage ~35%), and region 3 of normal-appearing brain tissue (nuclei coverage ~10%). Rows 2 and 3 of the figure show 20× magnified sections from within each ROI (red box on the full H&E image). H&E sections are displayed on row 2 and DAPI stained sections on row 3. The ROIs were carefully selected from relevant regions of the brain, and the magnification clearly demonstrates the difference in cellular density between each ROI. Note that the MRIs and the histological slides are not coregistered, only adjusted geometrically and resliced accordingly. The ROIs extracted from the histological slides do not represent a 1:1 selection of regions between the two. Hence, the histological slides are not used for qualitative measurements in the study, and the regions should only be viewed as a quantitative verification based on relative measurements.

The *T*_2,low_ were coregistered to *T*_2,high_ using cubic interpolation. The raw DW data were corrected for spatial and intensity distortion caused by the B0 magnetic field inhomogeneities using the reversing gradient method (38). The EPI-corrected DW images were then coregistered to *T*_2,low_ and further to the high resolution space using the same coregistration matrix as applied between *T*_2,low_ and *T*_2,high_. Finally, the coregistered DW images and the *T*_2,high_ images were resliced according to the histology space using cubic interpolation. We did not fully coregister the histology and MR data due to the low resolution and low number of slices of the DW images compared to that of the histology. Hence, histology slides were used for visual and quantitative validation only. The resliced MRI slices and histology slides used in the study are presented in Figure 1. Subsets of DWI data were generated by averaging all directional diffusion data to avoid directional dependency and normalized to the corresponding b0 acquisition to exclude differences in *T*_2_ weighing across the data schemes from the analysis. Subtraction maps were created by subtracting the normalized EPI-corrected averaged signal image acquired at Δ = 11 ms from the image acquired at Δ = 60 ms for each *b*-value.

Tissue ROIs were drawn based on histology (cell density) and structural *T*_2_-maps. Two MRI slices were chosen for the analysis, and a set of high, intermediate, and low cellular density ROIs were drawn on both MRI slices and histologic slide separately; the high cell density tumor region (region 1, Figures 1A,B) of primary tumor cells (~18.8 mm^3^, ~50% nuclei coverage), the intermediate density tumor region (region 2, Figures 1A,B) near the injection site (~7.0 mm^3^, ~35% nuclei coverage), and the low density region of normal-appearing brain tissue (NAB, region 3, Figures 1A,B), extracted from the healthy part of the mouse brain (~28.2 mm^3^, ~10% nuclei coverage). All ROIs were drawn in regions of high SNR (i.e., the superior half of the brain, near the surface coil). All histological regions had a cell body size distribution ranging between 3 and 12 μm. As the MRIs and H&E slides could only be paired based on visual interpretation, the estimated nuclei coverage and cell size distribution are only evaluated as a quantitative approximation in ROIs in the MRI images and, therefore, not included in the following analysis.

To separate the restricted and hindered diffusion signal, we used a biexponential model for the normalized DW-signal [*S*_n_ = *S*/*S*(TE)]

(3) $$S_{\text{n}}=f\cdot \text{exp}(-b\cdot {\text{ADC}}_{\text{r}}\text{(}\Delta \text{,}R\text{))+}\left(1-f\right)\cdot \text{exp(}-b\cdot {\text{ADC}}_{\text{h}}\text{)}$$

where *f* is the restricted DW-signal fraction, ADC_h_ and ADC_r_ are the hindered (extracellular) and restricted (intracellular) ADC. To avoid over fitting, and due to the small data set, we used literature values for the λ and *D*_ex_, as these parameters have been widely studied previously. The ADC_h_ was defined as ADC_h_ = *D*_ex_/λ^2^, with λ = 1.73 for the high-density tumor region and 1.46 < λ < 1.73 for intermediate-density tumor region (14), and *D*_ex_ ~ 3 μm^2^/ms (4). ADC_r_ was modeled for water diffusion restricted within spheres of radius *R* using Δ = 11–60 ms and *b* = 0–4000 s/mm^2^ as variables, assuming no exchange between extra and intracellular compartment (20):

(4) $$\begin{array}{l} {\text{ADC}}_{r}\left(\Delta ,R\right)=\frac{2}{D_{\text{intra}}\delta^{2}\left(\Delta -\frac{\delta }{3}\right)}\cdot \sum_{\text{m}=1}^{\infty }\frac{\alpha_{\text{m}}^{-4}}{\alpha_{\text{m}}^{2}R^{2}-2}\\ \left[2\delta -\frac{1}{\alpha_{\text{m}}^{2}D_{\text{intra}}}\cdot \left(\begin{array}{l} 2+\text{exp}\left[-\alpha_{\text{m}}^{2}D_{\text{intra}}\left(\Delta -\delta \right)\right]-2\text{exp}\left[-\alpha_{\text{m}}^{2}D_{\text{intra}}\delta \right]\\ -\;2\text{exp}\left[-\alpha_{\text{m}}^{2}D_{\text{intra}}\Delta \right]+\text{exp}\left[-\alpha_{\text{m}}^{2}D_{\text{intra}}\left(\Delta +\delta \right)\right] \end{array}\right)\right] \end{array}$$

*R* is the cell radius, *D*_intra_ is the intrinsic intracellular diffusivity previously measured at 1 μm^2^/ms (16, 17), α_m_ is the m-th root of $\left(\alpha R\right)\cdot {J^{\prime }}_{3/2}^{\;}\left(\alpha R\right)-\frac{1}{2}J_{3/2}\left(\alpha R\right)=0$, and *J* is the Bessel function. By combining Eqs 3 and 4, cellular radius *R*, ADC_r_, and cellular signal fraction (*f*) were estimated in a voxel-wise analysis for both tumor ROIs using non-linear modeling and minimizing the sum of squares. As the normal tissue had very low SNR at high *b*-value, the reduced model in Eq. 3 with constant ADC_r_ was used to model the NAB data. Note also that *f* is not a direct measure of cellular fraction in this model but is weighted by *T*_2_ relaxation (Eq. 1), which may differ between the two diffusion pools (24).

## Results

The Δ-dependence on the restricted DW-signal was measured using DW data acquired with multiple Δ at various *b*-values in a cb experiment.

**Figure 2:**
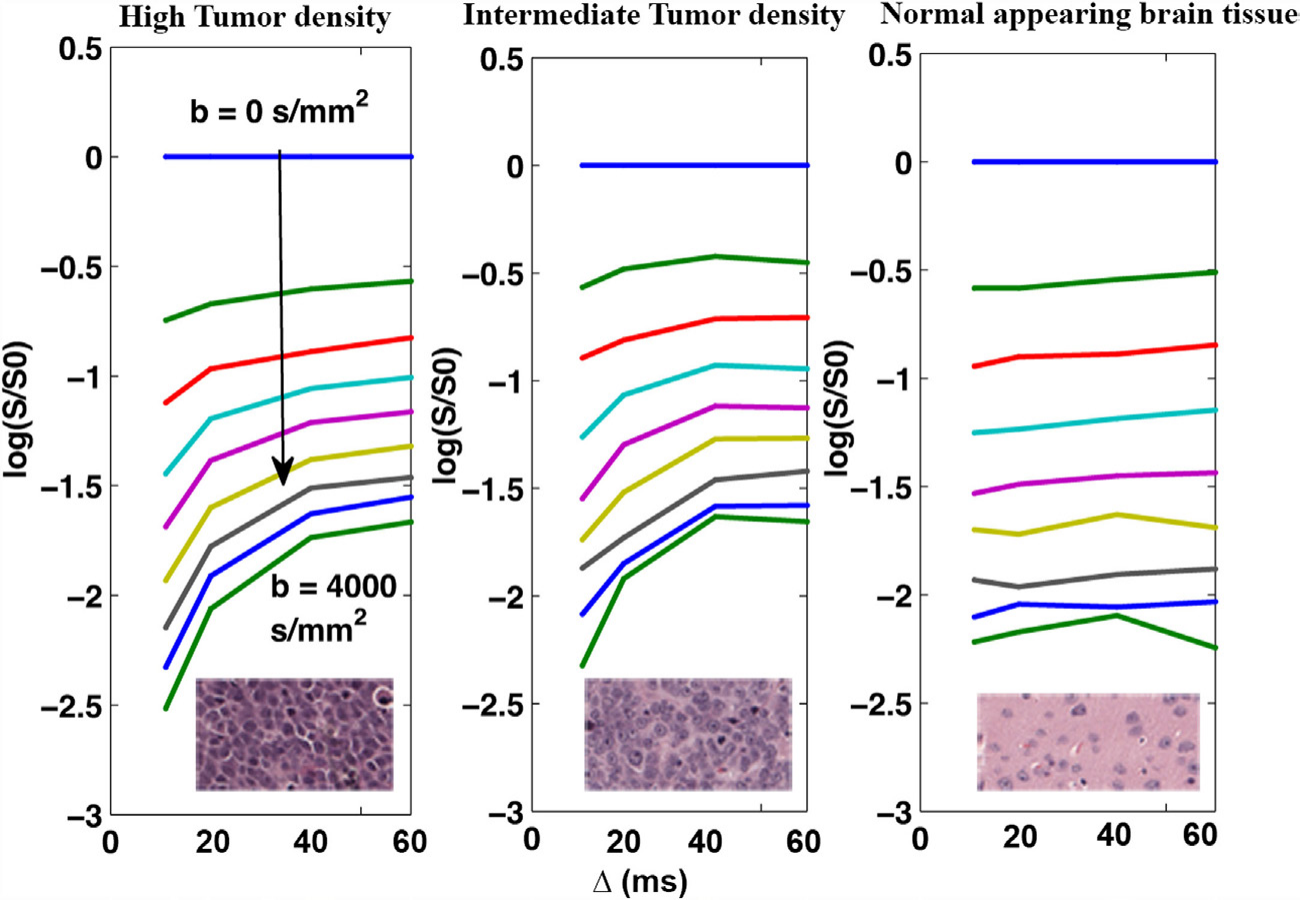
**Mean DW-signal as a function of Δ for the three tissue regions averaged across the two MRI slices**. The DW-signal (plotted on log scale) from both the primary tumor site with high cell density (left) and the intermediate region with intermediate cell density (middle) demonstrate strong dependence on Δ, with 140% increase in signal intensity over the range of 10–60 ms.

Figure 2 shows the average DW-signals (both slides added together) plotted as a function of Δ for the three tissue regions displayed in Figures 1A,B. As shown, signals from both intermediate- and high-density tumor regions demonstrate strong Δ-dependency (140% signal increase in both regions at *b* = 4000 s/mm^2^), whereas NAB signal demonstrate minimal dependency of Δ (0% signal increase). The Δ-dependency of the tumor regions was strongest with high *b*-values, indicating that the effect is related to the restricted DW-signal, as predicted.

**Figure 3:**
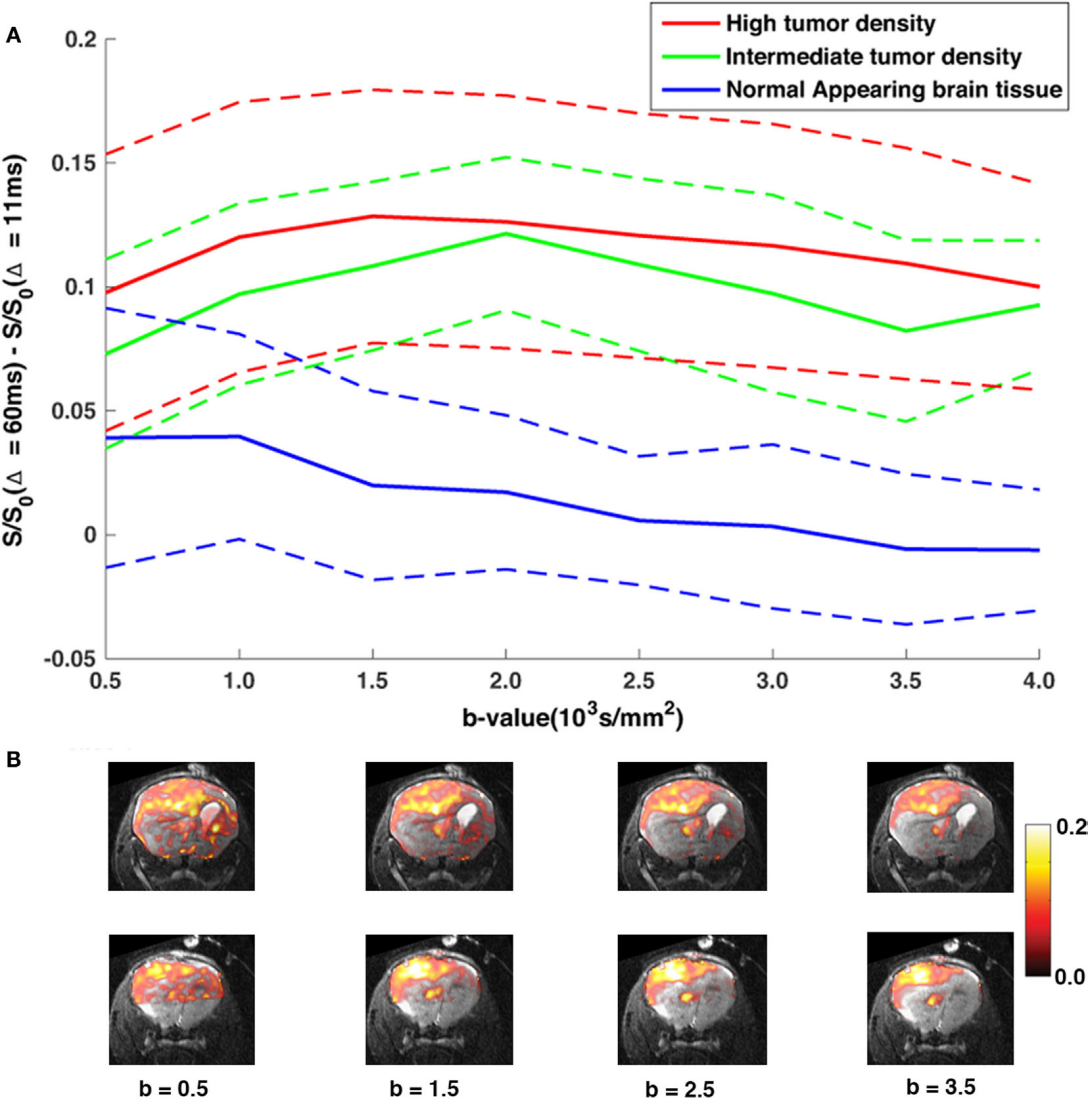
**The DTD contrast with increasing *b*-value**. **(A)** The average DTD contrast (solid lines) as a function of *b*-value for each ROI, averaged across both MR slices, with corresponding SD (dashed lines) **(B)** DTD-maps overlaid *T*_2,high_ images of two MRI slices analyzed in the study as a function of *b*-value. As predicted, the contrast between the NAB regions and tumor regions is weighted by the amount of restricted diffusion in the diffusion signal, and our results show that, at *b* > 1500 s/mm^2^, tumor tissue signal is well separated from the NAB signal and remains constant with increasing diffusion weighing, even within regions with lower tumor cell densities.

By subtracting the DW-images acquired at Δ = 11 ms from images acquired at Δ = 60 ms, we obtained maps of non-Gaussian diffusion signals [“diffusion time-dependent” (DTD) contrast]. Figure 3 show the effect of increasing the *b*-value on the DTD contrast in terms of signal contrast within each ROI (Figure 3A) and subtraction maps (Figure 3B). As predicted, the contrast between the NAB regions and the tumor regions is based on the amount of restricted diffusion in each region and is constant at diffusion weighing of *b* = 1500 s/mm^2^ and higher.

The biexponential model was used to extract quantitative parameter estimates and verify the interpretation of the DTD contrast. The parameter estimates can be found in Table 1, and plots can be found as Supplementary Material. Generally, the results show a good fit to the real data, and the signal decay of all tissue regions were consistent with a fast and a slow diffusion component. The NAB region demonstrated no Δ-dependency and was modeled using a reduced biexponential model, whereas the slow DW-signal component from both tumor regions were highly dependent on Δ in tumor regions, consistent with the gradual fill-up of restricted water within the intracellular space.

**Table 1:** **Results of the biexponential modeling of restricted and hindered DW-signal (Eqs 3 and 4)**.

	High tumor density	Intermediate tumor density	Normal-appearing brain tissue
*R* (μm)	5.2 ± 0.2	3.7–5.9 ± 1.7	–
*F*	0.31 ± 0.10	0.20–0.35 ± 0.10	0.56 ± 0.05
ADC_h_ (mm^2^/ms)	1	1–1.4	1.9 ± 0.29
ADC_r_ (mm^2^/ms)			0.41 ± 0.04
Δ = 11 ms	0.41 ± 0.04	0.26–0.30 ± 0.06	
Δ = 20 ms	0.28 ± 0.04	0.15–0.18 ± 0.05	
Δ = 40 ms	0.15 ± 0.03	0.08–0.09 ± 0.02	
Δ = 60 ms	0.09 ± 0.04	0.05–0.09 ± 0.04	
λ	1.73	1.73–1.46	1.26 ± 0.10

*Here, only tumor tissue signal was modeled using Δ-dependent ADC, as NAB was only weakly influenced by the restricted diffusion and demonstrated no Δ-dependence. A reduced biexponential model (Eq. 3) was used for modeling of the NAB signal*.

## Discussion

By studying the DW-signal of various diffusion times in a constant *b*-value experiment, we have shown that the slow diffusion component of the DW-signal is non-Gaussian, consistent with restricted diffusion of the intracellular space.

Multiple previous studies suggest that the restricted DW-signal is sensitive to tumor presence, and separation of the slow ADC from fast ADC improves tumor contrast-to-noise and reduces sensitivity to extracellular edema when compared to standard (extracellular) ADC DWI (31–34). Our current understanding of the basic underlying mechanism associated with the slow diffusion component is limited, and most previous studies, which investigate the biexponential signal behavior in tissue, model the DW-signal based on complex parameter fitting procedure and *a priori* assumptions (6, 16, 20, 21, 28–30, 40). The proposed method in the presented study, however, provides a simple technique for isolation of the restricted DW-signal component and serves as a complimentary and direct approach for separating the restricted from the hindered diffusion effects. Because the hindered and free DW-signals are weighted by Gaussian diffusion and thus independent of Δ, subtraction of cb-data acquired at long and short Δ compared to the squared compartment size (*d*^2^) isolates the DTD-dependent, non-Gaussian, restricted DW-signal component directly. The technique allows for quantification of DTD-restricted diffusion without a model and parameter fitting.

By applying the proposed method, we found that the tumor tissue signal was Δ-dependent during the Δ-interval chosen for our study and largely weighted by non-Gaussian diffusion. In contrast, signal from the NAB regions was measured with no Δ-dependence and dominated by hindered, Gaussian diffusion and non-DTD restricted diffusion. The observed DTD contrast within both tumor regions remained constant at *b* > 1500 s/mm^2^, consistent with intracellular restriction. As the extracellular space of the intermediate tumor density tissue has large unrestricted regions similar to that of the normal brain tissue (Figure 1), yet, is characterized by a strong DTD contrast, it is reasonable to assume that the observed contrast arise from restricted water “filling up” the intracellular space during Δ [as previously suggested (24)]. Following this assumption, the difference in tissue cell density between the tumor and the normal tissue regions may partly explain the prominent difference of Δ-dependency on the DWI-signal within these regions, as a higher cell density yields an increased restricted signal contribution (more cell volume) to the overall DW-signal. In addition, as the Δ-dependence is compartment size-dependent, the normal tissue regions may (this was not measured in our study) have a higher number of larger or smaller cellular compartments, which do not experience the gradual molecular “fill-up” of the intracellular space during Δ compared to that of tumor tissue. These assumptions are also consistent with the histology, which showed a distribution of cell sizes within each region. Therefore, we argue that the reported reduced ADC in high-grade tumor tissue (2, 3, 41) may be a result of a heavier weighting on the restricted intracellular signal in these tumor regions compared to normal brain tissue regions due to both increased cell packing and tissue cell size. The biexponential modeling of the NAB data supports this claim, as we found evidence of a slow ADC component in these regions as well, but without the DTD contrast.

By investigating the signal curve of Figure 2, we may estimate an approximate cellular compartment size directly, as the elbow seen in plots Figures 2A,B imply that water molecules start approaching the restricted fill-up regime at Δ = 40 ms. The minimum diffusion length scale of the tumor tissue should in this case be on the order of *d*^2^ = 2 ⋅ *D*_intra_ ⋅ 40 ms. If we now use the previously measured intracellular diffusivity *D*_intra_ ~ 1 μm^2^/ms (16, 17), this approximates to *d* ~ 9 μm, which is comparable to the estimates found using the two-compartment model of *d* ~ 11 μm and within the distribution range of cell body size estimate on histology *d* ~ 3–12 μm. It is also in agreement with some previous estimates of the glioma cell size (16, 24). The importance of restricted diffusion on tumor detection, and the influence of intracellular ADC in cancer imaging, is supported by the results of the biexponential fit, where we were able to replicate reasonable diffusion and compartment size values for both the intermediate and tumor tissue. The signal volume fraction was, however, slightly underestimated within the tumor regions compared to the approximated cell coverage of the histological regions. This discrepancy may be explained by a difference in T2-signal weighing of the intracellular and extracellular regions, which was not accounted for in our model (24). In addition, as the DTD is cell size-dependent, the signal fraction, estimated based on our model, may simply reflect the signal contribution from cells experiencing the molecular fill-up of the intracellular space rather than reflecting the true cell density of the region. An experiment including more and shorter Δ-values may possibly have improved the output of the extended biexponential model.

Despite being largely attenuated at high *b*-values, the output from the modeling of the NAB signal attenuation suggests a significant contribution from a slow diffusion component from this region, which may add support to the assumption that the slow ADC is intracellular. The signal fraction in these estimates was, however, largely overestimated compared to the space fraction occupied by cells on the NAB-section of the histological images. Considering the low SNR at high *b*-values in these regions, the overestimation may simply be a consequence of a high noise level rather than reflecting the signal contribution of intracellular restricted diffusion.

The estimations using the proposed model for restricted diffusion were highly dependent on the chosen λ, with decreasing *R* with decreasing λ. This represents a limitation in the parameter estimations in the model.

Although the presented research is a simple and novel approach for studying the restricted DW-signal directly, and the focus in our work was to show that the slow ADC is non-Gaussian and a consequence of intracellular restriction, there are still a few limitations, which should be addressed. In the current empirical study, we were unable to measure restricted diffusion in the normal tissue, hence the proposed differences between the normal and tumor regions related to the DTD contrast remains hypothetical. Furthermore, the reproducibility of the fill-up phenomenon using other tumor types and validating the DTD effect in regions with higher degree of heterogeneity or within tumor tissue in an early stage of development is left unexplored in this study. Specifically, considering the observed difference in Δ-dependence between normal and cancer tissues, extending the analysis of the DTD contrast to include a wider range of tumor tissue populations would be an interesting next step in order to test the applicability of the DTD contrast and its potential as biomarker of cancer. Changing the gradient amplitude/Δ may create differences in eddy current artifacts across the volumes (42). These artifacts typically manifest as geometric distortions including shearing, shading, scaling, blurring, and spatial misregistration of the data. As the subtraction maps between the two did not display evident blurring or edge effects, which would be expected as a result of misregistration and differences in shearing, we argue that differences in eddy current effects are small compared to the strong Δ-effect and should not contribute significantly to the outcome of our study. This was, however, not tested specifically.

The present study was performed on a specialized high-performance animal MRI scanner. Utilizing short Δ is not currently available on clinical scanners, however, clinical translation of the principle may still be achievable on next generation human clinical scanners with greater gradient performance (43).

## Conclusion

In this empirical study, we verify the assumption that the slow diffusion component of the DW-signal is non-Gaussian and dependent on Δ, consistent with restricted diffusion of the intracellular space. The importance of restricted diffusion in tumor detection is consistent with our results.

## Author Contributions

TH: contributed to conception, acquisition, analysis, and interpretation of data, drafting, and writing the article. NW: contributed to conception, acquisition, analysis, and interpretation of data and revising the article. JK: contributed to conception, acquisition, and analysis of data and participated in revising the article. YC: contributed to acquisition of data and participated in revising the article. GY: contributed to acquisition of data and participated in drafting and revising the article. HB: contributed to analysis of data and participated in revising the article. NS-A: contributed to acquisition of data and participated in revising the article. RR-P: contributed to conception, acquisition, analysis, and interpretation of data and revising the article. RB: contributed in acquisition of data and participated in revising the article. NN: contributed to acquisition of data and participated in revising the article. SK: contributed to interpretation of data and participated in revising the article. AB: contributed to conception, acquisition, and interpretation of data and participated in revising the article. AD: contributed to conception, acquisition, and interpretation of data and drafting and revising the article.

## Conflict of Interest Statement

The authors declare that the research was conducted in the absence of any commercial or financial relationships that could be construed as a potential conflict of interest. The reviewer SK and handling editor declared their shared affiliation, and the handling editor states that the process nevertheless met the standards of a fair and objective review.

## Abbreviations

Δ, diffusion time; cb, constant *b*-value; DTD, diffusion time-dependent; NAB, normal-appearing brain; RSI, restriction spectrum imaging.
