# Supplementary figures and images for: Demonstration of Non-Gaussian Restricted Diffusion in Tumor Cells Using Diffusion Time-Dependent Diffusion-Weighted Magnetic Resonance Imaging Contrast
Source: Front Oncol. 2016 Aug 2;6:179. doi: 10.3389/fonc.2016.00179 (PMC4970563; doi:10.3389/fonc.2016.00179)

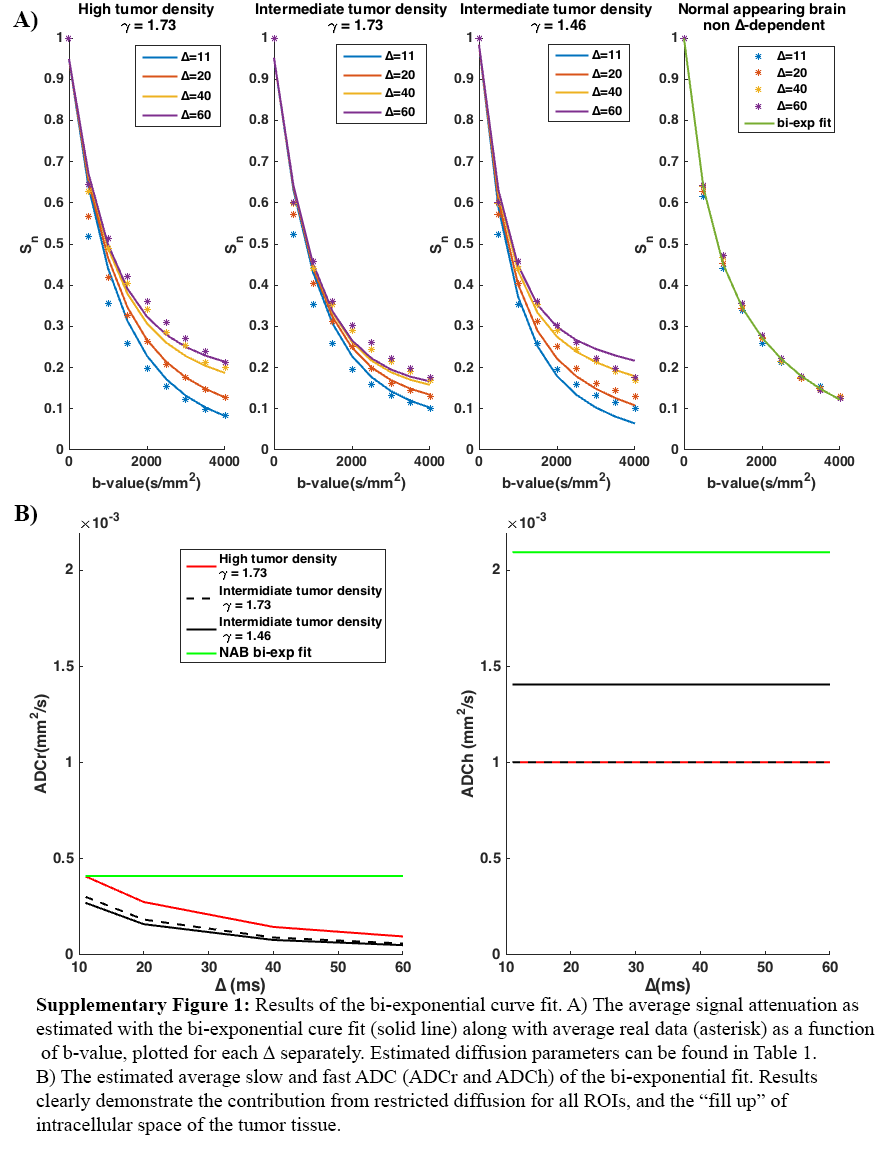

Supplement: Supplementary file 1 [file image_1.tif]
